# Microscopic Polyangiitis with Pulmonary Fibrosis: An Often-Recognized Manifestation of the Disease

**DOI:** 10.1155/2019/2673947

**Published:** 2019-12-31

**Authors:** Liam M. Clifford, Jamma Li, Christopher J. Renaud, Suran L. Fernando

**Affiliations:** ^1^Department of General Medicine, Gosford Hospital, Gosford, NSW, Australia; ^2^Department of Clinical Immunology and Allergy, Royal North Shore Hospital, Sydney, NSW, Australia; ^3^Immunorheumatology Laboratory, New South Wales Health Pathology, Royal North Shore Hospital, Sydney, NSW, Australia; ^4^University of Sydney, Sydney, NSW, Australia; ^5^Department of Anatomical Pathology, Royal North Shore Hospital, Sydney, NSW, Australia

## Abstract

**Background:**

Microscopic polyangiitis (MPA) can manifest with atypical features such as pulmonary fibrosis and chronic obstructive pulmonary disease (COPD), which are atypical and unusual features of small vessel vasculitis.

**Case Presentation:**

This paper presents two patients with microscopic polyangiitis and respiratory symptoms attributable to atypical pulmonary manifestations. Pulmonary fibrosis was present in both cases, with COPD also present in one patient. Management involved methylprednisone, prednisone, and cyclophosphamide. The second patient also received azathioprine. Both patients responded well to immunosuppressive treatment; however, pulmonary fibrosis and COPD were refractory to immunosuppression.

**Conclusion:**

Pulmonary manifestations including pulmonary fibrosis, emphysema, and bronchiectasis are observed in MPA. Evaluation of MPA in unexplained cases should be performed to avoid delays in diagnosis and management. Patients who present with MPA with pulmonary manifestations may respond to treatment, but their pulmonary features demonstrate a refractory nature to such management.

## 1. Background

Microscopic polyangiitis (MPA) as defined by the Chapel Hill Consensus Conference criteria 2012 is a pauci-immune necrotizing systemic small vessel vasculitis usually associated with the presence anti-neutrophil cytoplasmic antibodies (ANCA) and anti-myeloperoxidase (MPO) antibodies [1]. Other pauci-immune vasculitides include granulomatosis with polyangiitis (GPA) and eosinophilic granulomatosis with polyangiitis (EGPA). In contrast to these other entities, MPA does not involve granulomatous inflammation. The European Medicines Agency (EMA) algorithm [2] established the criteria for MPA by firstly excluding EGPA by either the American College of Rheumatology (ACR) criteria (4 of 6 criteria comprising asthma, >10% eosinophils on the differential leukocyte count, mononeuropathy (including multiplex) or polyneuropathy, migratory or transient pulmonary opacities detected radiographically, paranasal sinus abnormality, and biopsy containing a blood vessel showing the accumulation of eosinophils in extravascular areas) [3] or the Lanham criteria (presence of asthma, peak eosinophilia >1.5 × 10^9^/L, and systemic vasculitis in ≥2 extrapulmonary sites) [4]. Then, secondly excluding GPA by the CHCC definition (necrotizing granulomatous inflammation usually involving the upper and lower respiratory tract and necrotizing vasculitis affecting predominantly small to medium vessels) and the ACR criteria (presence of 2 of 4 criteria comprising nasal or oral inflammation, abnormal chest radiograph showing nodules, fixed infiltrates, or cavities, abnormal urinary sediment, granulomatous inflammation on biopsy of an artery or perivascular area, or positive ANCA in the absence of a biopsy). These algorithms do not reliably differentiate between GPA and MPA in all patients; the defining pathological difference between GPA and MPA is the presence of granulomatous changes on biopsy which may be missed due to the sampling error; there is overlap in the clinical features and in the ANCA serologies between GPA and MPA, and some patients present initially with manifestations consistent with MPA and subsequently develop features consistent with GPA. The ability to establish a diagnosis is important given the higher rate of relapse with PR3-ANCA vasculitis [5], and the different manifestations associated with each of the vasculitides such as subglottic stenosis in GPA [6] and interstitial lung disease (ILD) in MPA [7] may require different monitoring and therapies. The Diagnostic and Classification of the Systemic Vasculitides (DCVAS) study has proposed a scoring system for MPA with pauci-immune glomerulonephritis scoring +3, p-ANCA or anti-MPO antibody positive +6, fibrosis or interstitial lung disease (ILD) on chest imaging +3, bloody nasal discharge/ulcers/crusting/congestion or blockage/septal defect/perforation −3, c-ANCA or anti-PR3 antibody −1, and eosinophil count >1 × 10^9^/L −4 with a total score of ≥6 needed for classification with a sensitivity 87% and specificity 96% [8].

Despite the difficulties in classification, pulmonary disease is an important manifestation of MPA in addition to constitutional symptoms, glomerulonephritis, peripheral neuropathy, and skin rash. A retrospective chart review of 115 patients in a Western Spain multicentre survey revealed the presence of lung infiltrates in 28% of 74 individuals with MPO-ANCA disease and 16% of 51 individuals with MPA as classified by EMA [9].

Some forms of interstitial lung disease in MPA include pulmonary fibrosis with or without emphysema and are considered atypical manifestations and are usually resistant to conventional therapy for ANCA-associated vasculitis (AAV) [7, 10].

We describe two cases of MPA with pulmonary fibrosis, one with accompanying emphysema to illustrate the pertinent clinical, laboratory, and histological features and the response to treatment.

## 2. Case Presentation

### 2.1. Case One

**Figure 1:**
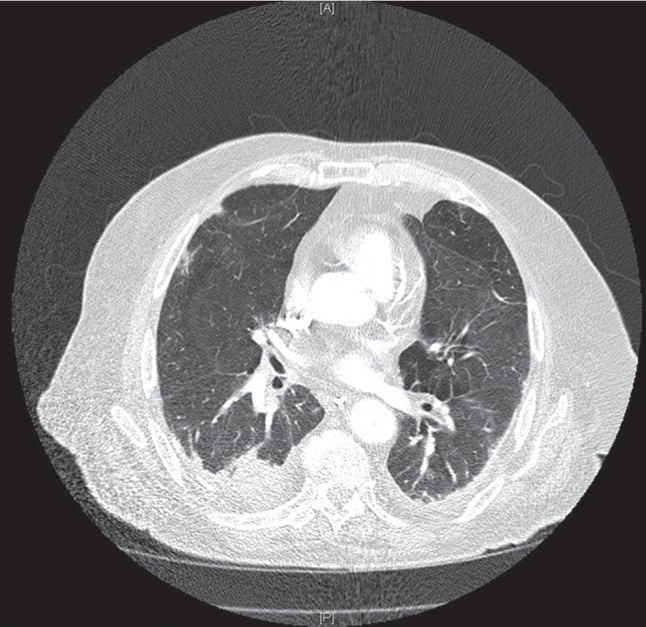
An axial CT chest image of patient 1 demonstrating long-standing bilateral fibrosis and nodular opacities with honey combing and traction bronchiectasis affecting the left lung.

A 64-year-old female presented with significant weight loss over several months (20 kg), a chronic dry cough, exertional dyspnoea, paraesthesiae in her feet and a left foot drop, erythematous maculopapular rash affecting her feet, and peripheral oedema. Her past medical history included hypertension, a previous occipital infarct, pulmonary fibrosis, falls, hypothyroidism, osteoarthritis, and osteopenia and was a previous smoker. There was no history of haemoptysis. Her CXR showed diffuse bronchiectasis in the left lung and the right perihilar region. A high-resolution CT scan demonstrated marked loss of the left lung volume, reduction in hemithorax size, left-sided honey combing, inter- and intralobular septal thickening, traction bronchiectasis, and mediastinal shift to the left in keeping with compensatory right lung hypertrophy. The right lung field also showed fibrotic changes with peripheral nodules affecting predominantly the left upper lobes but also evident in the right middle and lower lobes and diffuse interlobular and intralobular septal thickening with traction bronchiectasis more prominent in the left than right lung. HRCT findings were consistent with usual interstitial pneumonia (UIP) according to the American Thoracic Society (ATS) and the European Respiratory Society (ERS) guidelines (Figure 1).

**Figure 2:**
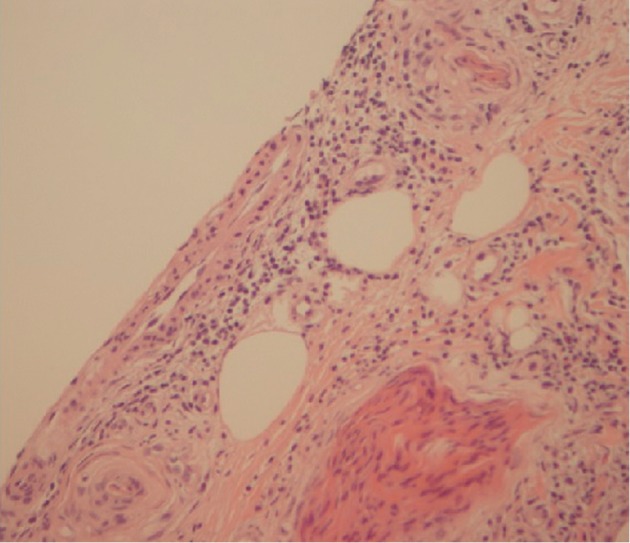
A sural nerve biopsy showing subcutaneous vessels with leukocytoclasis, fibrinoid necrosis, and a perivascular lymphocytic infiltrate (H&E).

MRI and angiography of the brain demonstrated punctuate foci of an elevated diffusion signal within the left basal ganglia and the splenium of the corpus callosum as well as the deep temporal and frontal white matter, suggesting a vasculitic process. Additionally, an MRI spine showed loss of normal high signal intensity within the L4/L5 and L5/S1 intervertebral disc spaces, consistent with a degenerative process without nerve root or cord encroachment. Cerebrospinal fluid (CSF) analysis showed normal levels of protein and glucose, an absence of white cells, no culture growth, and no oligoclonal bands. Subsequent nerve conduction studies revealed a severe length-dependent sensorimotor axonal neuropathy, while a sural nerve biopsy demonstrated fibrinoid necrosis and a lymphocytic infiltrate but no evidence of granulomas (Figure 2). Initial respiratory function tests revealed severe restrictive disease with a forced expiratory volume in one second (FEV1)/forced vital capacity (FVC) ratio of 1.15. Her FEV1 was 39.6%, FVC was 37.9%, total lung capacity (TLC) was 45.2%, and diffuse lung capacity for binding carbon monoxide (DLCO) of 40.9% was predicted, respectively. A transthoracic echocardiogram (TTE) showed normal systolic function, no significant valvular disease, and an ejection fraction (EF) of 65%. The subsequent transoesophageal echocardiogram revealed no mural thrombus or signs of pulmonary hypertension. A skin biopsy of the rash demonstrated deep perivascular lymphocytic inflammation with no deposition of immunoglobulins or complement on direct immunofluorescence.

Laboratory investigations revealed a C-reactive protein (CRP) level of 250 mg/L (<5), erythrocyte sedimentation rate (ESR) 84 mm/hr (<15), and perinuclear-ANCA (p-ANCA) (titre 80) with elevated anti-myeloperoxidase (MPO) antibodies of 20 U/mL (<6). Her full blood count (FBC) including the eosinophil count (0.2 × 10^9^/L) was normal. The renal function and urine albumin-creatinine ratio were normal. A diagnosis of microscopic polyangiitis was established with the atypical pulmonary manifestation of pulmonary fibrosis/UIP.

The patient was commenced on intravenous (IV) pulse methylprednisolone 750 mg daily for three days followed by a tapering course of oral prednisone commenced at 1 mg/kg daily. She underwent seven cycles of IV cyclophosphamide over six months before switching to a maintenance therapy of azathioprine 150 mg daily. Prednisone was completely weaned after twelve months. Her maintenance treatment was complicated by an episode of atypical community-acquired pneumonia. She responded to a course of IV ceftriaxone and azithromycin followed by oral amoxicillin/clavulanic acid. Subsequently, the azathioprine was reduced to 100 mg daily. After four years of azathioprine therapy, she remained in remission with near-complete resolution of her left foot drop. A recent anti-MPO antibody level was 10 U/ml. Nerve conduction studies demonstrated resolution of the axonal sensorimotor neuropathy. Her progress CT scans of the chest revealed no change in her pulmonary fibrosis and bronchiectasis. Progress respiratory function tests revealed a persistent restrictive pattern with an FEV1/FVC of 91.1 but with an improvement in the FEV1 of 53.6% and FVC of 46.3% predicted, respectively. This appeared consistent with the resolution of her pulmonary nodules. The TLC and DLCO were unchanged. Her progress TTE was normal, in particular there has been no changes suggestive of pulmonary hypertension. Her azathioprine will be slowly weaned over the next year.

### 2.2. Case Two

**Figure 3:**
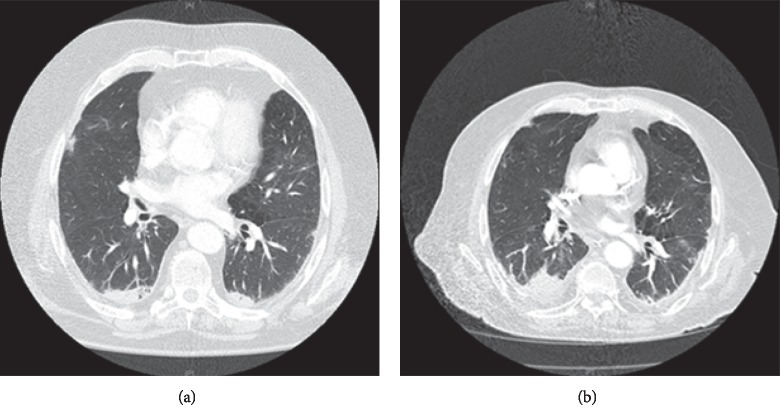
(a) CT pulmonary angiogram of patient 2 reveals bilateral calcified pleural plaques, subpleural fibrosis, paraseptal emphysematous change, and a small region of honey combing in the right lower lobe base laterally. (b) CT chest six months later revealing interval increase in the size of the consolidation, resolution of the right upper lobe spiculated lesions, and stable pulmonary fibrosis.

**Figure 4:**
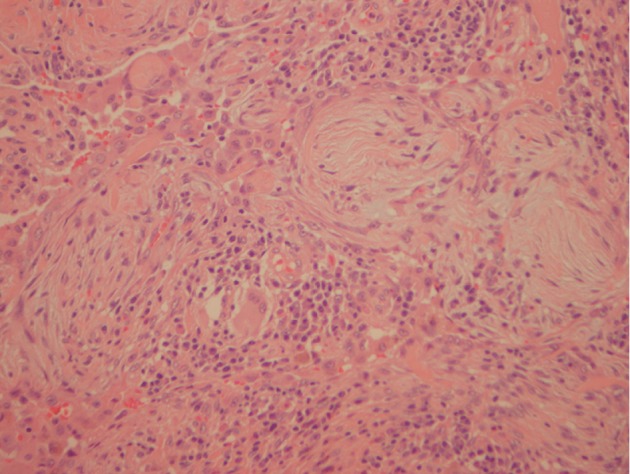
Left lung biopsy of patient two showed subpleural proliferative fibroblastic foci and a nodular infiltrate of plasma cells, histiocytes, and multinucleated giant cells.

A 69-year-old male presented with a six-month history of dyspnoea, anorexia, weight loss (40 kg), and a glove-and-stocking paraesthesia. His past medical history included chronic obstructive pulmonary disease (COPD), ischaemic heart disease, hyperlipidaemia, hypertension, hyperthyroidism, a thyroid nodule, a previous pulmonary embolus, L4/L5 laminectomy, and iron-deficiency anaemia. The patient was an ex-smoker and had previous significant environmental exposure to asbestos. Prior to admission, his colonoscopy was normal whilst a CXR and CT pulmonary angiogram (CTPA) revealed several small (up to 8 mm) spiculated lesions in the right upper lobe, bilateral calcified pleural plaques, bilateral subpleural fibrosis, bilateral subpleural emphysematous change, and a small region of honey combing in the right lower lobe base laterally (Figure 3(a)). HRCT findings were consistent with UIP (ATS/ERS guidelines) and in combination with bronchiectasis, a finding of combined IPF and emphysema (CPFE) was noted. A core biopsy of the right lung lesion demonstrated a mixed inflammatory infiltrate with no evidence of malignancy or asbestos bodies, but cytology revealed giant cells. A CT chest six months later revealed resolution of the spiculated lesions of the right upper lobe, but increase in subpleural fibrosis parenchyma was noted in the previous scan (Figure 3(b)). Respiratory function tests at this time revealed a FEV1/FVC ratio of 1.04, indicating severe restrictive lung disease. A TTE showed normal systolic function, no significant valvular disease, and an EF of 55%. There were no changes suggestive of pulmonary hypertension. The biopsy of the left lung pleura demonstrated macronodular collections of plasma cells, lymphocytes, and histiocytes with histiocytic multinucleated giant cells and foci of young fibrous nodules. No necrotizing granulomas were evident. There were changes of emphysema with plentiful intra-alveolar pigmented histiocytes (Figure 4). Direct immunofluorescence stains were negative for immunoglobulins and complement.

Laboratory investigations revealed a positive p-ANCA (titre 160) and MPO antibodies 90 U/mL (<6). He had normal levels of anti-proteinase 3 antibodies at 2 IU/mL (<6). The remainder of his autoimmune serology: antinuclear, extractable antigen nuclear, antidouble-stranded DNA, anticitrullinated peptide antibodies, and rheumatoid factor and his complement levels (C3, C4) was normal. His CRP level was 58 mg/L (<5) and ESR was 106 mm/hr (<15). FBC showed anaemia with a haemoglobin level of 114 g/L (135–160) and ferritin of 1512 ng/mL (30–300). His eosinophils were normal (0.4 × 10^9^/L).

His CT of the sinuses showed pansinusitis characterised by mucosal thickening without adjacent bone erosion. There was also suspected renal involvement due to 90% dysmorphic red cells in the context of normal renal function with a protein-creatinine ratio of 19 mg/mmol (<30) and a normal renal tract ultrasound.

A diagnosis of AAV was established based on pulmonary giant cell inflammation, dysmorphic haematuria, peripheral neuropathy, and constitutional symptoms in the absence of malignancy. The atypical feature of CPFE was noted on presentation.

Initial management was IV pulse methylprednisone 1 g daily for three days followed by a five-month tapering course of oral prednisone commenced at 1 mg/kg. IV cyclophosphamide was initiated (15 mg/kg every four weeks for six months). Following the completion of the prednisone, he was commenced on azathioprine 150 mg daily.

He responded well to treatment with resolution of his constitutional symptoms and peripheral neuropathy. A repeat CT chest performed following cyclophosphamide therapy and three months of azathioprine therapy revealed resolution of parenchymal airspace opacities but stable pleural plaques. He had a normal repeat TTE. There was normalisation of CRP, anti-MPO antibody, haemoglobin, and ferritin levels.

## 3. Discussion

Our two cases demonstrated the clinical, laboratory, and histological features of MPA with accompanying atypical pulmonary disease.

Diffuse alveolar haemorrhage secondary to pulmonary capillaritis is the most frequently described respiratory manifestation of MPA [11]. However, PF is becoming increasingly appreciated as a manifestation of the disease. A Greek study showed 39% of 33 patients with MPA followed for a median period of 13 months had PF with only one of these patients developing this feature after presentation [7]. A retrospective review over 15 years of an Argentinian cohort with MPA revealed 9 of 28 (32%) patients had PF. Five of these patients had clinical symptoms related to their fibrosis prior to the development of their manifestations of vasculitis by up to 108 months [12]. In a retrospective study of 61 consecutive Japanese patients with an initial diagnosis of IPF, MPO-ANCA was positive in 3 patients (5%) and MPO-ANCA positive conversion occurred in 6 patients (10%), of whom 2 developed manifestations of MPA [13]. In a Japanese retrospective study of 31 patients with pulmonary fibrosis and MPO-ANCA, the histopathological features were analyzed in 15 patients with 13 patients demonstrating interstitial fibrosis extending from the alveolar walls to the proximal interstitial tissues and interlobular septa. Honeycomb lesions were detected in 12 cases. Evidence of vasculitis was found in 8 cases [14], but this finding was not evident in another Japanese study consisting of 9 patients [13].

The pathogenesis of IPF is yet to be fully elucidated, but a number of possibilities exist. Recurrent alveolar haemorrhage including subclinical alveolar bleeding due to pulmonary capillaritis could result in pulmonary fibrosis [15]. MPO-ANCA may play a direct role in the pathogenesis of pulmonary fibrosis. Oxidative stress through the production of hypochlorous acid from the interaction of MPO with anti-MPO antibodies may trigger the fibrosis. IPF may induce ANCA during the chronic inflammation process as a result of neutrophil destruction accounting for the extent of IPF seen at presentation of MPA [7]. There is speculation that tobacco smoke exposure may be involved in the pathogenesis of PF by activating and stimulating MPO expression on epithelial cells. A Japanese retrospective study of 9 patients with PF and MPO-ANCA were all smokers [13]. Exposure to tobacco leads to macrophage activation and neutrophil recruitment, with consequent elastin and collagen breakdown, and subsequent accelerated matrix destruction and emphysema [16]. A Japanese study multicentre longitudinal retrospective CT imaging study comprising 150 hospital patients with MPA revealed 7% of patients had PF and 4% had CPFE [17]. Although 34% of this cohort had a history of smoking, it is unclear whether this to the PF and CPFE patients. However, Cottin et al. showed that combined PF and emphysema (CPFE) occurs independently of smoking in connective tissue diseases such as rheumatoid arthritis and systemic sclerosis [18]. CPFE, characteried by dyspnoea, upper-lobe emphysema, lower-lobe fibrosis, and abnormalities of gas exchange, is complicated by pulmonary hypertension, acute lung injury, and lung cancer and associated with high mortality rates (median survival rates 2.1 to 8.5 years) [19]. One-year survival rate for those with pulmonary hypertension confirmed by right heart catheterization is 60% [20]. IPF without coexistent emphysema confers a worse prognosis. In a Japanese retrospective study of 31 patients with pulmonary fibrosis and MPO-ANCA, the 5-year survival rate of 50% was comparable to those with idiopathic pulmonary fibrosis and worse than patients with MPO-ANCA-negative pulmonary fibrosis with collagen vascular diseases [14]. The causes of death include progressive respiratory failure and its complications (pulmonary haemorrhage, and pneumonia) as well as lung cancer. PF increases the risk of lung malignancy, mainly non-small-cell lung cancer, by 7–20% [21].

Current EULAR/ERA-EDTA recommendations for the induction of remission in organ-threatening AAV involve high-dose corticosteroids and IV cyclophosphamide or rituximab for three to six months [22]. Once remission is induced, azathioprine, methotrexate, or rituximab is continued for a minimum of 2 years. The recent findings of the RITAZAREM randomized control study involved 170 patients with AAV who experienced a relapse and then achieved remission during 4 months of induction therapy with rituximab and corticosteroids, and rituximab demonstrated that maintenance with rituximab 1 g every 4 months was superior in the prevention of relapse than azathioprine 2 mg/kg daily (13 vs. 38%) [23]. Specific therapy for AAV patients with PF is not well established, and further studies are required. A French retrospective multicentre study including 49 patients with AAV and PF demonstrated that the 3-year survival rate in patients initially treated with cyclophosphamide or rituximab combined with corticosteroids was superior to patients treated with corticosteroids alone (94% vs. 64%, *P*=0.03) [24]. It remains to be determined whether pirfenidone, an antifibrotic agent that inhibits transforming growth factor-beta-stimulated collagen synthesis, extracellular matrix deposition, and fibroblast proliferation, or nintedanib, a receptor blocker for multiple tyrosine kinases that mediate elaboration of fibrogenic growth factors, will be efficacious in these patients. These agents appear to slow disease progression [25, 26] and decrease mortality rates [27, 28] in patients with IPF.

This report comprises two patients, but we aim to alert clinicians to the atypical pulmonary features of MPA that are chronic, resistant to conventional immunosuppression, and are associated with a poor prognosis due to progressive respiratory insufficiency and associated complications such as pulmonary haemorrhage, pneumonia, and lung cancer. Further studies are required to elucidate optimal treatment for patients with AAV and their treatment resistant-pulmonary manifestations.

## 4. Conclusion

MPA presents with a systemic and nonspecific symptom and can mimic other vasculitides. Laboratory tests including ANCA are useful for evaluating suspected vasculitis. It is important that a confirmatory tissue diagnosis of AAV is established. Pulmonary manifestations including fibrosis, CPFE, and bronchiectasis are frequently associated with MPA, but the underlying mechanisms require further elucidation. Recognition of these manifestations as a feature of the patient's vasculitis may avoid a delay in the diagnosis and implementation of appropriate therapy. Such manifestations are treatment-resistant to conventional immunosuppression for AAV, and further studies are required to determine other treatment modalities to reverse these changes in symptomatic patients.

## Abbreviations

AAV: ANCA-associated vasculitides
ANCA: Anti-neutrophil cytoplasmic antibodies
COPD: Chronic obstructive pulmonary disease
CPFE: Combined pulmonary fibrosis and emphysema
CRP: C-reactive protein
CT: Computed tomography
CXR: Chest X-ray
DLCO: Diffusion capacity for carbon monoxide
EF: Ejection fraction
EGPA: Eosinophilic granulomatosis with polyangiitis
ESR: Erythrocyte sedimentation rate
FBC: Full blood count
FEV1: Functional expiratory volume in 1 second
FVC: Functional vital capacity
GPA: Granulomatosis with polyangiitis
IV: Intravenous
MPA: Microscopic polyangiitis
MPO: Myeloperoxidase
MRI: Magnetic resonance imaging
p-ANCA: Perinuclear anti-neutrophil cytoplasmic antibodies
TLC: Total lung capacity
TTE: Transthoracic echocardiogram.
